# Novel Anticoagulants for Stroke Prevention in Atrial Fibrillation: A Systematic Review of Cost-Effectiveness Models

**DOI:** 10.1371/journal.pone.0062183

**Published:** 2013-04-23

**Authors:** Brendan L. Limone, William L. Baker, Jeffrey Kluger, Craig I. Coleman

**Affiliations:** 1 Department of Pharmacy Practice, University of Connecticut School of Pharmacy, Storrs, Connecticut, United States of America; 2 Department of Pharmacy, Hartford Hospital, Hartford, Connecticut, United States of America; 3 Department of Cardiology, Hartford Hospital, Hartford, Connecticut, United States of America; Universidad Peruana de Ciencias Aplicadas (UPC), Peru

## Abstract

**Objective:**

To conduct a systematic review of economic models of newer anticoagulants for stroke prevention in atrial fibrillation (SPAF).

**Patients and Methods:**

We searched Medline, Embase, NHSEED and HTA databases and the Tuft’s Registry from January 1, 2008 through October 10, 2012 to identify economic (Markov or discrete event simulation) models of newer agents for SPAF.

**Results:**

Eighteen models were identified. Each was based on a lone randomized trial/new agent, and these trials were clinically and methodologically heterogeneous. Dabigatran 150 mg, 110 mg and sequentially-dosed were assessed in 9, 8, and 9 models, rivaroxaban in 4 and apixaban in 4. Warfarin was a first-line comparator in 94% of models. Models were conducted from United States (44%), European (39%) and Canadian (17%) perspectives. Models typically assumed patients between 65–73 years old at moderate-risk of stroke initiated anticoagulation for/near a lifetime. All models reported cost/quality-adjusted life-year, 22% reported using a societal perspective, but none included indirect costs. Four models reported an incremental cost-effectiveness ratio (ICER) for a newer anticoagulant (dabigatran 110 mg (n = 4)/150 mg (n = 2); rivaroxaban (n = 1)) vs. warfarin above commonly reported willingness-to-pay thresholds. ICERs vs. warfarin ranged from $3,547–$86,000 for dabigatran 150 mg, $20,713–$150,000 for dabigatran 110 mg, $4,084–$21,466 for sequentially-dosed dabigatran and $23,065–$57,470 for rivaroxaban. Apixaban was found economically-dominant to aspirin, and dominant or cost-effective ($11,400–$25,059) vs. warfarin. Indirect comparisons from 3 models suggested conflicting comparative cost-effectiveness results.

**Conclusions:**

Cost-effectiveness models frequently found newer anticoagulants cost-effective, but the lack of head-to-head trials and the heterogeneous characteristics of underlying trials and modeling methods make it difficult to determine the most cost-effective agent.

## Introduction

Atrial fibrillation (AF) affects approximately 3 million people in the Unites States (U.S.), and this number may reach as high has 12 million by 2050 [1]. AF is associated with a significant financial burden, costing the U.S. healthcare system about $26 billion annually [2]. While hospitalizations are the primary driver of these costs (52%); the cost of pharmacologic management of AF is also noteworthy (23%) [3].

One of the primary concerns accompanying the diagnosis of AF is the associated 4- to 5-fold increase in ischemic stroke risk [4]. Guidelines for the management of AF recommend the use of pharmacologic agents for the prevention of stroke depending on baseline risk [5]–[7]. For patients at moderate-to-high risk of stroke, a vitamin K antagonist such as warfarin has traditionally been recommended. However, its use has been limited by its narrow therapeutic index and food and drug interactions [8], [9]. Therefore, alternative anticoagulants have been evaluated in recent years. To date, two agents (dabigatran, rivaroxaban) have received approval by the United States Food and Drug Administration (FDA) for prevention of stroke and systemic embolism in patients with AF, with a third (apixaban) currently under consideration. Clinical trials have demonstrated these agents to have at least similar impact on reducing stroke rates compared to warfarin with comparable or improved safety profiles [10]–[12].

An important step in determining the place of these newer anticoagulants in clinical practice is to evaluate their cost-effectiveness. This fact is highlighted by the discussion of cost-effectiveness data (although not exhaustive) in recent national guidelines for pharmacologic stroke prevention in AF (SPAF) [7]. Numerous economic models have been published to evaluate the cost-effectiveness of these newer oral anticoagulants for SPAF [13]–[30]. Accordingly, we undertook a systematic review of economic models of dabigatran, rivaroxaban and apixaban for SPAF.

## Patients and Methods

### Data Sources and Searches

We searched the MEDLINE, EMBASE, National Health Service Economic Evaluation Database (NHS EEDS) and Health Technology Assessment (HTA) bibliographic databases along with the Tufts Cost-Effectiveness Analysis Registry. Searches were conducted for economic studies published between January 2008 and October 10, 2012. The start date of our search corresponded with the first published outcomes study of dabigatran. Our searches utilized Medical Subject Heading (MeSH) terms and keywords for AF, economic modeling and the newer anticoagulants (**see Text S1**). Finally, we also reviewed references from included models to identify additional relevant citations.

### Study Selection

Two investigators independently reviewed all abstracts and screened all potentially relevant, full-text articles for inclusion in a parallel manner using *a priori*-defined criteria. We included evaluations of the cost-effectiveness of pharmacologic agents for SPAF using a Markov or discrete event simulation model design. To be included models had to evaluate both cost (in monetary units) and effectiveness outcomes (i.e., life-years or quality-adjusted life-years (QALYs)). Models had to be available as a full-text publication and be published in the English language. Manufacturer’s models reported as part of government reports [i.e., National Institute for Health and Clinical Excellence (NICE) or Canadian Agency for Drugs and Technologies in Health (CADTH)] were also included in this review; however, models presented solely at professional meetings or available only in abstract form were excluded.

### Data Extraction

Two investigators used a standardized data abstraction tool to independently extract data for each model with disagreement resolved by discussion. We collected the following information from each model: 1) primary comparisons made; 2) characteristics of the base-case population; 3) model structure and assumptions (e.g., similarity to “progenitor” models, health states, study perspective, discount rate, time horizon, cycle length, types of sensitivity analysis, willingness-to-pay threshold(s) (WTP(s)) utilized etc.); 4) characteristics related to both internal and external of the models themselves and that of the randomized trials underlying/driving the economic models (e.g., use of blinding, intention-to-treat methods, inclusion/exclusion criteria, CHADS_2_ scores, methods for dosing warfarin, time in the therapeutic international normalized ratio (INR) range, etc.); and 5) results including base-case and sensitivity analyses. For the purpose of this review, a “progenitor” model was defined as the earliest published models using a distinct structure and serving as a template for future models.

### Quality Assessment of Economic Models and Underlying Trials

We conducted a critical appraisal of the methodology and reporting of the included models (with the exception of the government reports) using the Quality of Health Economic Studies (QHES) rating scale [31], [32]. The QHES is a validated assessment of quality for cost-effectiveness analyses and contains 16 evaluable items. Each item carries a weighted point value, with total possible scores ranging from 0 (lowest quality) to 100 (highest quality). An explanation of our QHES scoring of included models is available in **Supporting Information: Text S2**. In addition, we evaluated the internal validity of the models’ “underlying” trials using the Jadad scale [33]. For the purpose of this review, “underlying” trial(s) were defined as those used as the principal sources for drug-specific safety and efficacy inputs in each of the economic analyses. The Jadad scale assesses inherent controllers of bias by assessing randomization, double-blinding, and proper reporting of patient withdrawals. These individual components were assessed and an aggregate score was calculated for each included trial (0 = weakest, 5 = strongest). Two investigators performed all quality assessments independently with disagreement resolved through discussion.

### Data Synthesis

The current report provides summary statistics and qualitative (descriptive) synthesis of identified economic models in the form of tables and figures. Categorical data are reported as percentages, while continuous data are reported as means ± standard deviations. The authors have followed the PRISMA Statement in reporting this systematic review (**see Checklist S1**).

## Results

**Figure 1:**
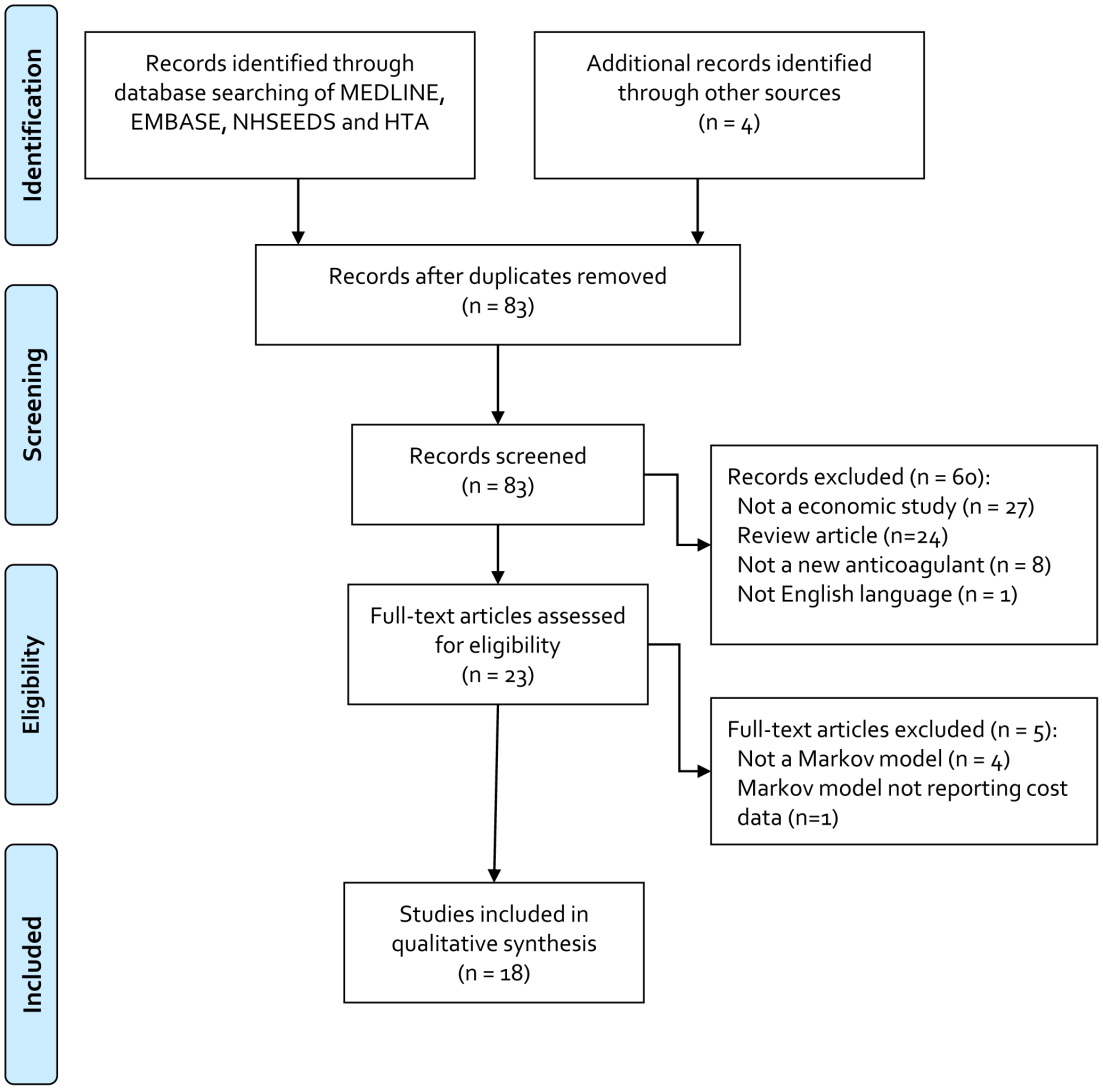
Results of Literature Search.

The literature search initially identified 83 non-duplicate citations (**Figure 1**). Upon title and abstract review, 60 citations were excluded, leaving 23 articles for full-text review. Upon full-text review, 5 articles were excluded, leaving a total of 18 models for inclusion in our systematic review (**Table 1**) [13]–[30].

**Table 1:** Characteristics of Included Models.

Author, Year	Primary Comparisons	Characteristics of Base-Case Population	Basic Model Structure	Time Horizon (Years)/Cycle length(Months)	Reported Perspective	Discount Rate	Drug Persistence	Funding	QHES
**Dabigatran**									
Freeman, 2011 [13]	Dabigatran 110 mgDabigatran 150 mgAdjusted-dose warfarin	65 year old with AF and CHADS2 score ≥1 and no CI to anticoagulation	Gage	35/0.5	SocietalUS	3%	Any major hemorrhage (ICH or major ECH) resulted in cessation of anticoagulation therapy and initiation of ASA	F, G	78
Pink, 2011 [14]	Dabigatran 110 mgDabigatran 150 mgSequential dabigatranAdjusted-dose warfarin	71.5 year old with AF and a mean CHADS2 score of 2.1, 32.4% with a CHADS2≥3 and no CI to anticoagulation	Discrete event simulation	Lifetime/NA	UK NHS	3.5%	Patients who discontinued dabigatran because of a bleed or who discontinued warfarin (for any reason) were switched to aspirin. Patients who discontinued dabigatran for reasons other than bleeds were switched to warfarin. Assumed the rates of discontinuation of treatment in the second year of the RE-LY study (21% of dabigatran and 17% of warfarin patients) persisted for the lifetime of treatment	G	70
Shah, 2011 [15]	Dabigatran 110 mgDabigatran 150 mgAdjusted-dose warfarinClopidogrel+ASAASA	70 year old with AF and CHADS2 score of 1–2 and no CI to anticoagulation	Gage	20/1	MedicareUS	3%	If stroke/TIA occurred, patient was switched to dabigatran 150 mg. If major bleed occurred while taking warfarin or dabigatran, patients were switched to ASA, if patients were on ASA they discontinued treatment. If stroke and major bleed occurred, patients reinitiated initial treatment. Discontinuation rate = 20% after 24 months	F	75
Sorensen, 2011 [16]	Dabigatran 110 mgDabigatran 150 mgSequential dabigatran^a^Adjusted-dose warfarin “Real-world prescribing”	69 year old with AF with ischemic stroke risk matched to RE-LY population (mean CHADS2 score of 2.1) and no CI to anticoagulation, 20% had prior TIA/stroke	Sorensen	Lifetime/3	Canadian Ministry of Health	5%	50% permanently stopped all treatment after non-fatal ECH. Discontinuation of anticoagulation could occur due to event, such as GI symptoms (dyspepsia) or burden of anticoagulation clinic or poorly controlled INR	P	78
Spackman, 2011 [17] (Manufacturer’s model)	Dabigatran 110 mgDabigatran 150 mgSequential dabigatranAdjusted-dose warfarinClopidogrel+ASAASA	71 year old with AF and CHADS2 score of 1–2 and no CI to anticoagulation	Sorensen	Lifetime/3	UK NHS	3.5%	Clinical events leading to permanent treatment discontinuation included hemorrhagic stroke and ICH. ECH was assumed to result in permanent discontinuation for 50% of the patients	P, G	NA
Davidson, 2012 [18]	Sequential dabigatranAdjusted-dose warfarin	65 year old with AF with a CHADS2 risk matched to RE-LY	Unclassified	20/12	SocietalSweden	3%	Patients who discontinue warfarin switch to ASA or no treatment in equal proportions. Patients who discontinue dabigatran switch to warfarin, ASA, or no treatment in equal proportions. Themodel assumes that the proportion of patients who discontinue treatment decreases at the same rate as during the first 2 years of RE-LY	P, G	77
Gonzalez-Juanatey, 2012 [19]	Sequential dabigatranAdjusted dose warfarinReal-world prescribing in Spain (60% VKA, 30% ASA, 10% no therapy)	69 year old with AF with ischemic stroke risk matched to RE-LY population (mean CHADS2 score of 2.1)	Sorensen	Lifetime/3	Spanish National Health System	3%	Patients who experience an ICH or hemorrhagic stroke discontinue the treatment permanently. After experiencing an ECH, patients can discontinue the treatment temporally (50% of the cases during a 3–month cycle) or permanently (the remaining 50%). In cases of permanent treatment discontinuation for reasons other than the development of ischemic stroke or ICH, 70% of the patients change to a second-line treatment regimen	NR	81
Kaml, 2012 [20]	Dabigatran 150 mgAdjusted-dose warfarin	70 year old with AF with a prior stroke or TIA and no CI to anticoagulation	Gage	20/1	SocietalUS	3%	Assumed patients who developed ICH stopped anticoagulationand began lifelong aspirin therapy, whereas patients with a majorECH resumed anticoagulation after 1 month	NR	75
Kansal, 2012 [21]	Sequential dabigatranAdjusted-dose warfarinASANo treatment	69 year old with AF with ischemic stroke risk matched to RE-LY population (mean CHADS2 score of 2.1) and no CI to anticoagulation, 20% had prior TIA/stroke	Sorensen	Lifetime/3	UK Healthcare Perspective	3.5%	All hemorrhagic events could lead to discontinuation of treatment and patients could also discontinue treatment for non-clinical reasons. When discontinuing warfarin or dabigatran, ASA was administered. If ASA was discontinued, patients received no anticoagulation	P	89
Langkilde, 2012 [22]	Sequential dabigatranAdjusted-dose warfarin	69 year old with AF with ischemic stroke risk matched to RE-LY population (mean CHADS2 score of 2.1) and no CI to anticoagulation, 20% had prior TIA/stroke	Sorensen	20/3	Danish Healthcare Perspective	2%	Discontinue treatment if ICH occurred. ECH lead to permanent discontinuation in 50% of occurrences. Discontinuation could occur due to other adverse events, based on rates from RE-LY	P	68
You, 2012 [23]	Dabigatran 110 mgDabigatran 150 mgAdjusted dose warfarin (TTR = 64%)Adjusted dose warfarin (genotype-guided, TTR = 78.9%)	65 year old with AF and CHADS2 score of 2 or higher	Gage	25/1	PayerUS	3%	Patients surviving ischemic stroke would change the initial anticoagulation therapy to dabigatran 150 mg BID. Patients surviving any major bleeding event discontinued current anticoagulation and started on ASA alone	None	89
**Rivaroxaban**									
Lee, 2012 [24]	RivaroxabanAdjusted-dose warfarin	65 year old at high risk for stroke (CHADS2 score of 3) and no CI to anticoagulation	Gage	35/1	MedicareUS	3%	Major hemorrhagic events led to transition from rivaroxaban or warfarin to ASA	None	86
**Apixaban**									
Kamel, 2012 [25]	ApixabanAdjusted-dose warfarin	70 year old with AF with a prior stroke or TIA and no CI to anticoagulation	Gage	20/1	SocietalUS	3%	Assumed patients who developed ICH stopped anticoagulationand began lifelong aspirin therapy, whereas patients with a majorECH resumed anticoagulation after 1 month	NR	82
Lee, 2012 [26]	ApixabanAdjusted-dose warfarin	65 year old with AF and CHADS2 score of 2 and no CI to anticoagulation	Gage	Lifetime/0.5	MedicareUS	3%	Major hemorrhage warranted discontinuation of apixaban or warfarin and initiation of ASA	None	86
Lee, 2012 [27]	ApixabanASA	70 year old with AF, CHADS2 score of 2 and low risk of bleeding	Gage	1,10/1	MedicareUS	3%	Major hemorrhagic events led to transition from apixaban to ASA	None	86
**Dabigatran** **vs. Rivaroxaban**									
Edwards, 2011 [28](Manufacturer’s model)	RivaroxabanPooled dabigatran 110/150 mgSequential dabigatranAdjusted-dose warfarinASA	Patients with AF based on the population included in the ROCKET AF trial	Sorensen	Lifetime/3	UK NHS	3.5%	Minor/major ischemic stroke, systemic embolism and minor/major ECH resulted in temporary discontinuation of therapy. ICH resulted in permanent discontinuation of primary therapy in those with CHADS2≤2 and temporary discontinuation in those with CHADS2 of 3 or higher	P, G	NA
Kansal, 2012 [29]	RivaroxabanSequential dabigatranAdjusted-dose warfarin	73 year old patient with AF and CHADS2 risk matched to ROCKET-AF (mean CHADS2 score of 3.5)	Sorensen	Lifetime/3	Canadian Ministry of Health	5%	Anticoagulation was permanently discontinued or ASA initiated after ICH, while the discontinuation rate following an ECH event was estimated to be 10%. A total of 70% of dabigatran- and rivaroxaban-treated patients (and 78% of warfarin treated patients in the secondary analysis) who discontinued due to other reasons were assumed to switch to ASA	P	78
**Dabigatran vs. Rivaroxaban vs. Apixaban**									
Wells, 2012 [30]	RivaroxabanDabigatran 110 mgDabigatran 150 mgApixabanAdjusted-dose warfarin	Canadians with non-valvular atrial fibrillation with typical patient profile from RE-LY (72 years with no previous stroke or MI)	Sorensen	40/3	Canadian Ministry of Health	5%	Patients who have a ICH or major ECH while on warfarin, rivaroxaban, dabigatran or apixaban continue on treatment with aspirin alone	G	NA

AF = atrial fibrillation; ASA = aspirin; CI = contraindication; ECH = extracranial hemorrhage; F = foundation; G = government; GI = gastrointestinal; ICH = intracranial hemorrhage; INR = international normalized ratio; MI = myocardial infarction; NA = not applicable; NR = not reported; NHS = National Health Service; P = pharmaceutical company; RE-LY = Randomized Evaluation of Long-Term Anticoagulation Therapy; ROCKET-AF = Rivaroxaban Once Daily Oral Direct Factor Xa Inhibition Compared with Vitamin K Antagonism for Prevention of Stroke and Embolism Trial in Atrial Fibrillation; QHES = Quality of Health Economic Studies; TIA = transient ischemic attack; TTR = time in therapeutic range; UK = United Kingdom; US = United States; VKA = vitamin k antagonist.

a Sequential dabigatran = 150 mg BID in those <80 years of age and 110 mg BID in those >80 years of age.

All of the analyses were Markov models except one [14], which was a discrete event simulation. The majority of Markov models appeared to be derivatives of one of 2 earlier models created to assess the cost-effectiveness of adjusted-dose warfarin [16], [34]. Authors utilizing these “progenitor” models by Gage and Sorensen as templates made small modifications; such as the inclusion of myocardial infarction or dyspepsia as a health state [15], [23], or the alteration of the method for handling recurrent strokes [15], but preserved the core design of the models. A noteworthy difference between the two basic model structures is Sorensen’s inclusion of both ischemic stroke and systemic embolism as health states, which more closely matches the FDA-approved indication of the newer anticoagulants (**see Supporting Information: Figure S1**).

Included models reflected the healthcare systems of various countries, including eight from the U.S. [13], [15], [20], [23]–[27], four from the United Kingdom [14], [17], [21], [28], three from Canada [16], [29], [30], and one each from Denmark [22], Sweden [18] and Spain [19]. Patients, with a CHADS_2_ score generally between 2–3 (ranging from 0–6, often with percentages of the cohort at varying stroke risks to match the RE-LY [10] or ROCKET-AF [11] populations), initiated anticoagulant therapy between 65 and 73 years of age and were followed for as little as one year and up to a lifetime. Warfarin and dabigatran were the most common treatment arms, used in 94% and 78% of included models (**see** **Figure S2**), respectively, and dabigatran versus warfarin (56%) was the most frequent primary comparison (**see Figure S3**). Greater than two thirds of the warfarin containing models tested the impact of varying INR control on the reported results. There was a lack of consensus regarding drug persistence after acute events. After experiencing an intracranial hemorrhage (ICH), patients typically permanently discontinued anticoagulation and may or may not have initiated aspirin monotherapy, whereas after a non-fatal extracranial bleed, patients either temporarily discontinued treatment for up to 3 months before restarting the initial anticoagulant or permanently discontinued therapy. Drug discontinuation rates were typically derived from the underlying randomized controlled trial (RCT). Just under a quarter of models reported using a societal perspective, though none included indirect costs due to lost productivity. Cycle lengths ranged from two weeks to one year, with the most common being three months (44%). Costs and health outcomes were generally discounted appropriately using country-specific guidance at rates ranging from 2%–5%. Finally, just over one third of included models were funded or supported by pharmaceutical companies with other models receiving funding from government institutions and foundations.

The quality of the included models, using the QHES tool, ranged from a low of 68 [22] to a high of 89 [21], [23]. Thirteen of the 18 models (72%) had a QHES score >75 and were considered high quality. The most common reasons for lower quality scores on the QHES included incorrectly reporting the perspective used (i.e., claiming a societal perspective but not including indirect costs) or not justifying the chosen perspective; not conducting or describing a literature search to identify model inputs; failure to report or justify the discount rate used; not including health states such as minor bleeding or dyspepsia in the model (when relevant); and not providing information regarding model funding/sponsorship (**see Supporting Information: Figure S4**). All of the included models were strongly based upon/driven by at least one of 4 randomized controlled trials, or in the case of the few models comparing the cost-effectiveness of newer anticoagulants head-to-head, through an indirect statistical comparison of these same trials [10]–[12], [35]. **Table 2** includes detail from the clinical trials that “underlie” the reviewed models, including quality scoring for each. Of note all but one trial [10], which utilized an open-label design to compared dabigatran vs. warfarin, scored a five on the Jadad scale.

**Table 2:** Characteristics of Underlying Trials.

Study, Year(N)	DrugComparator	Design Features	Mean CHADS2score	Duration	Ischemic StrokeRate (%/Year)	Major Bleeding Rate (%/Year)	Intracranial Bleeding Rate (%/Year)	Minor Bleed Rate (%/Year)	MI Rate (%/Year)	Quality Score
RE-LY,2009N = 18,113 [10]	Dabigatran 110 mg BIDDabigatran 150 mg BIDAdjusted-dose warfarin (TTR = 64%)	R, OL^a^, ITT	2.1	Median follow-up 2 years	1.340.921.20	2.713.113.36	0.230.300.74	13.1614.8416.37	0.720.740.53	3(2,0,1)
AVERROES, 2011N = 5,599 [35]	Apixaban 5 mg BIDAspirin 81–324 mg	R, DB, ITT	2.0	Mean follow-up 1.1 years	1.13.0	1.41.2	0.40.4	6.35.0	0.80.9	5(2,2,1)
ROCKET-AF, 2011N = 14,264 [11]	Rivaroxaban 20 mgAdjusted-dose warfarin (TTR = 55%)	R, DB, ITT	3.5	Median follow-up 707 days	1.7‡2.2‡	3.63.4	0.50.7	11.3 b11.1^b^	0.91.1	5(2,2,1)
ARISTOTLE, 2011N = 18,201 [12]	Apixaban 5 mg BIDAdjusted-dose warfarin (TTR = 62.2%)	R, DB, ITT	2.1	Median follow-up 1.8 years	0.971.05	2.133.09	0.330.80	15.97 c22.71^c^	0.530.61	5(2,2,1)

BID – twice daily; DB = double blind; ITT = Intention to Treat; MI = Myocardial Infarction; N = Population of Study; OL = Open Label; R = Randomized Trial; TTR = Time in Therapeutic Range.

a Double-blinding was used in RE-LY, but only for the dabigatran arms. Since the corresponding Markov model compared the cost-effectiveness of dabigatran (both doses) to warfarin, we report this trial as “open-label” above;

b Difference between rate of major or clinically relevant nonmajor bleeds and major bleeds.

c Difference between reported any bleed and reported major bleed rates;

‡ Stroke or systemic embolism;

### Dabigatran Models

**Figure 2:**
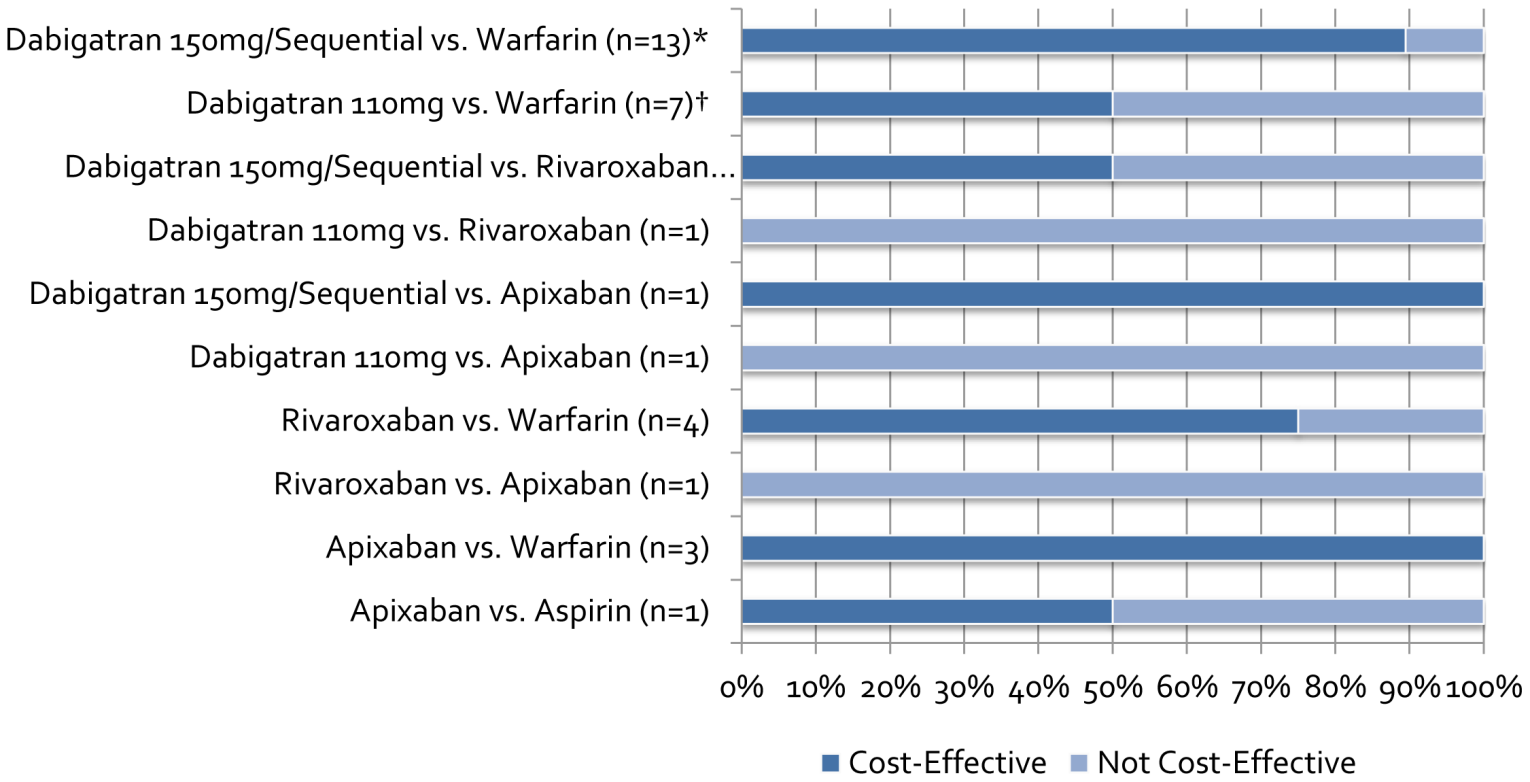
Proportion of Reported Incremental Cost-Effectiveness Ratios Below Reported Willingness-to-Pay Threshold. *Includes results of dabigatran compared to “real-world prescribing”, “trial-like” warfarin control and genotype-guided warfarin †Includes results of dabigatran compared to “trial-like” warfarin control and genotype-guided warfarin.

Of the 13 models that directly compared dabigatran to warfarin, 8 assessed dabigatran 150 mg, 7 assessed dabigatran 110 mg, and 8 assessed sequentially-dosed dabigatran. Seven models based on the “progenitor” model by Sorensen et al. [16] were very similar in terms of model characteristics, with slight adjustments pertaining to specific countries (e.g., country-specific costs, discount rates, life tables to model non-event death). On the other hand, the four models based on Gage et al. [34] had more variation in model properties and structure (e.g., time horizon, cycle length, population characteristics, health states modeled). Of note, one model based on Gage et al. included only patients with a prior stroke or transient ischemic attack (TIA) [20], while the other models included a mixed population of AF patients with or without a prior stroke or TIA (typically around 20%). Of the remaining two models, one employed discrete event simulation, and the other exhibited a unique model structure. All 13 dabigatran models included a myocardial infarction (MI) health state, 11 included a minor bleed health state, and 12 assessed the impact of INR control on the results. Eight of the 13 models included a systemic embolism health state (seven of which were derivatives of Sorensen et al.), but only two of 13 modeled a dyspepsia health state despite this adverse event significantly differing in incidence between treatment groups in RE-LY. All 13 models derived efficacy and safety data from the RE-LY trial. In total, 78% of dabigatran vs. warfarin ICERs were cost-effective at their respective WTP thresholds (four dabigatran 110 mg and two 150 mg comparisons vs. warfarin had ICERs above commonly reported WTPs) and ranged from $3,547–$86,000 for dabigatran 150 mg; $20,713–$150,000 for dabigatran 110 mg; and $4,084–$21,466 for sequentially-dosed dabigatran (**Table 3** **,** **Figure 2**). The model by Shah et al. [15] did not find dabigatran cost-effective, perhaps due to the chosen cost of dabigatran. The authors surveyed four retail pharmacies and used the median cost of USD$9 per day, whereas other models typically used a cost less than USD$5 per day. Freeman et al. [13] also utilized a higher cost for dabigatran which may have pushed the ICER for dabigatran 110 mg above the WTP threshold. Though dabigatran 150 mg was cost-effective in their original analysis, the authors updated the results based on a lower cost of dabigatran 150 mg which decreased the ICER from $43,372 to $12,386 compared to warfarin. Of the 13 models comparing dabigatran to warfarin, 9 performed probabilistic sensitivity analyses (PSA) which demonstrated dabigatran 150 mg to be cost-effective in 44.9%–93% of iterations; dabigatran 110 mg in 42%–67% of iterations; and sequentially-dosed dabigatran in 82%–100% of iterations at the lowest reported WTP threshold compared to warfarin. All 13 models performed one-way sensitivity analyses and the results were often sensitive to baseline rates/relative risks of ischemic stroke or ICH on dabigatran/warfarin, time in therapeutic INR range, and costs of acute events and long term disability care.

**Table 3:** Results of Included Models.

Author, Year	Primary Comparisons	Characteristics of Base-Case Population	ICER (Cost/QALY)									Sensitive or Influential Variables	MCS Results
**Dabigatran**													
Freeman, 2011 [13]	Dabigatran 110 mgDabigatran 150 mgAdjusted-dose warfarin	65 year old with AF and CHADS2 score ≥1 and no CI to anticoagulation	(2008 USD)**Compared to warfarin:**Dabigatran 110 mg$51,229Dabigatran 150 mg$45,372 ($12,386*)									Cost of dabigatran; stroke rate on warfarin and dabigatran; ICH rate on warfarin and dabigatran; utility on warfarin and dabigatran; utility after MI; monthly post-ICH cost	Dabigatran 150 mg was cost-effective 53% and 68% of the time compared to warfarin assuming a WTP = $50,000/QALY and $100,000/QALY, respectively
Pink,2011 [14]	Dabigatran 110 mgDabigatran 150 mgSequential dabigatranAdjusted-dose warfarin	71.5 year old with AF and a mean CHADS2 score of 2.1, 32.4% with a CHADS2≥3 and no CI to anticoagulation	(2009 GBP)**Compared to warfarin:**Dabigatran 110 mg£43,074 ($67,466 USD)Dabigatran 150 mg£23,082 ($36,153 USD)Sequential dabigatranNR				(2009 GBP)**Compared to dabigatran 150** **mg:**Dabigatran 110 mgDominated					Stroke rates on dabigatran or warfarin; vascular death rates on dabigatran or warfarin; increases to clinical event costs; drug utility losses	Dabigatran 150 mg was cost-effective 44.9% and 59.6% of the time compared to warfarin assuming WTPs = £20,000GBP/QALY and £30,000GPB/QALY, respectively.
Shah,2011 [15]	Dabigatran 110 mgDabigatran 150 mgAdjusted-dose warfarinClopidogrel+ASAASA	70 year old with AF and CHADS2 score of 1–2 and no CI to anticoagulation	(2010 USD)**Compared to warfarin:**Dabigatran 110 mg$150,000Dabigatran 150 mg$86,000Clopidogrel+ASADominated				(2010 USD)**Compared to ASA:**Dabigatran 110 mg$66,000Dabigatran 150 mg$50,000Warfarin$12,500Clopidogrel+ASA$99,000					Stroke rate; major bleed rate; time in INR range	NR
Sorensen, 2011 [16]	Dabigatran 110 mgDabigatran 150 mgSequential dabigatran aAdjusted-dose warfarin“Real-world prescribing”	69 year old with AF with ischemic stroke risk matched to RE-LY population (mean CHADS2 score of 2.1) and no CI to anticoagulation, 20% had prior TIA/stroke	(2010 CAD)**Compared to** **“trial-like” warfarin:**Sequential dabigatran $10,440 ($10,760 USD)	(2010 CAD)**Compared to** **“real-world prescribing”:**Sequential dabigatran$3,962($4,084 USD)			(2010 CAD)**Compared to** **“trial-like” warfarin:**Dabigatran 150 mg$9,041($9,319 USD)			(2010 CAD)**Compared to** **“trial-like” warfarin:**Dabigatran 110 mg$29,994($30,915 USD)		RR and rate of long-term disability of ischemic stroke on dabigatran; time in INR range; cost of INR monitoring; cost of disability care; time horizon	Dabigatran 150 mg and 110 mg were cost-effective 81% and 42% of the time compared to “trial-like” warfarin assuming a WTP = $30,000CAD/QALY. Sequential dabigatran was cost-effective 82% and 99% of the time compared to “trial-like” and “real-world” warfarin assuming a WTP = $30,000CAD/QALY
Spackman, 2011 [17]	Dabigatran 110 mgDabigatran 150 mgSequential dabigatranAdjusted-dose warfarinClopidogrel+ASAASA	71 year old with AF and CHADS2 score of 1–2 and no CI to anticoagulation	(2010 GBP)**Compared to warfarin:**Dabigatran 110 mg£18,680 ($30,048 USD)Dabigatran 150 mg£6,262 ($10,089 USD)Sequential dabigatran£13,157 ($21,198 USD)Clopidogrel+ASADominated				(2010 GBP)**Compared to ASA:**Dabigatran 110 mg£9,416 ($15,146 USD)Dabigatran 150 mg£4,441 ($7,156 USD)Warfarin£2,502 ($3,918 USD)Clopidogrel+ASADominated					Cost of dabigatran; baseline risk of ischemic stroke (CHADS2 score); ICH rate; time horizon	Dabigatran 150 mg was cost-effective 93% and 98% of the time compared to warfarin assuming WTPs = £20,000GBP/QALY and £30,000GPB/QALY, respectively. Dabigatran 110 mg was cost-effective 67% and 84% of the time compared to warfarin assuming WTPs = £20,000GBP/QALY and £30,000GPB/QALY, respectively
Davidson, 2012 [18]	Sequential dabigatranAdjusted-dose warfarin	65 year old with AF with a CHADS2 risk matched to RE-LY	(2010 EUR)**Compared to warfarin:**Sequential dabigatran€7,742 ($9,453 USD)									Baseline risk of ischemic stroke (CHADS2 score); time in INR range	Sequential dabigatran was cost-effective 100% of the time compared to warfarin assuming a WTP = €50,000EUR/QALY
Gonzalez-Juanatey, 2012 [19]	Sequential dabigatranAdjusted dose warfarinReal-world prescribing in Spain (60% VKA, 30% ASA, 10% no therapy)	69 year old with AF with ischemic stroke risk matched to RE-LY population (mean CHADS2 score of 2.1)	(2010 EUR)**Compared to warfarin**Sequential dabigatran€17,581 ($21,466 USD)				(2010 EUR)**Compared to real-world prescribing**Sequential dabigatran€14,118 ($17,224 USD)					Reduction in stroke risk; social costs (i.e., informal care, nursing home costs, institutional assistance, investments); INR control; cost of INR monitoring; time horizon	Dabigatran was cost-effective 96.4% and 99.9% of the time compared to warfarin and real-world prescribing, respectively, assuming WTP = €30,000EUR/QALY
Kamel,2012 [20]	Dabigatran 150 mgAdjusted-dose warfarin	70 year old with AF with a prior stroke or TIA and no CI to anticoagulation	(2010 USD)**Compared to warfarin:**Dabigatran 150 mg$25,000									Cost of dabigatran; relative risk of stroke on dabigatran; time in INR range; utility of mild ischemic stroke; monthly cost of stroke and ICH, patient age	Dabigatran 150 mg was cost effective 57% and 78% of the time compared to warfarin assuming WTPs = $50,000/QALY and $100,000/QALY, respectively
Kansal,2012 [21]	Sequential dabigatranAdjusted-dose warfarinASANo treatment	69 year old with AF with ischemic stroke risk matched to RE-LY population (mean CHADS2 score of 2.1) and no CI to anticoagulation, 20% had prior TIA/stroke	(2010 GBP)**Compared to warfarin:**Sequential dabigatran£4,831 ($7,566 USD)Sequential dabigatran (Age ≥80)£7,090 ($11,105 USD)			(2010 GBP)**Compared to ASA:**Sequential dabigatran£3,457 ($5,414 USD)			(2010 GBP)**Compared to no treatment:**Sequential dabigatranDominated			RR and baseline rates of ischemic and hemorrhagic stroke and ICH; time in INR range; cost of disability care; time horizon	Sequential dabigatran was cost-effective 98%, 100% and 100% of the time compared to warfarin, ASA and no treatment, respectively, assuming a WTP = £20,000GBP/QALY. Starting at 80 or above, sequential dabigatran was cost-effective 63% of the time compared to warfarin assuming a WTP = £20,000GBP/QALY
Langkilde, 2012 [22]	Sequential dabigatranAdjusted-dose warfarin	69 year old with AF with ischemic stroke risk matched to RE-LY population (mean CHADS2 score of 2.1) and no CI to anticoagulation, 20% had prior TIA/stroke	(2011 EUR)**Compared to warfarin:**Sequential dabigatran€6,950 ($8,739 USD)									Baseline risk of ICH; cost of INR monitoring	NR
You,2012 [23]	Dabigatran 110 mgDabigatran 150 mgAdjusted dose warfarin (TTR = 64%)Adjusted dose warfarin (genotype-guided, TTR = 78.9%)	65 year old with AF and CHADS2 score of 2 or higher	(2012 USD)**Compared to genotype-guided warfarin:**WarfarinDominatedDabigatran 110 mg$35,824Dabigatran 150 mg$13,810			(2012 USD)**Compared to warfarin:**Dabigatran 150 mg$3,547Dabigatran 110 mg$20,713			(2012 USD)**Compared to dabigatran 150** **mg:**Dabigatran 110 mgDominated			Time in INR range; utility of warfarin and dabigatran	Dabigatran 150 mg and 110 mg, genotype-guided warfarin and usual care warfarin were cost-effective 51.6%, 1.6%, 46.2% and 0.6% of the time assuming a WTP = $50,000/QALY
**Rivaroxaban**													
Lee,2012 [24]	RivaroxabanAdjusted-dose warfarin	65 year old at high risk for stroke (CHADS2 score of 3) and no CI to anticoagulation	(2011 USD)**Compared to warfarin:**Rivaroxaban$27,498									Cost of rivaroxaban; HR of stroke and ICH with rivaroxaban; utility with rivaroxaban; monthly cost of ICH; time horizon	Rivaroxaban was cost-effective 80.1% and 91.4% of the time compared to warfarin assuming WTPs = $50,000/QALY and $100,000/QALY, respectively
**Apixaban**													
Kamel, 2012 [25]	ApixabanAdjusted-dose warfarin	70 year old with AF with a prior stroke or TIA and no CI to anticoagulation	(2010 USD)**Compared to warfarin:**Apixaban$11,400									Cost of apixaban; rate of stroke on apixaban; rateof ICH; monthly cost of stroke and ICH; patient age	Apixaban was cost-effective in 62% and 81% of the time assuming WTPs of $50,000 and $100,000/QALY, respectively
Lee,2012 [26]	ApixabanAdjusted-dose warfarin	65 year old with AF and CHADS2 score of 2 and no CI to anticoagulation	(2011 USD)**Compared to warfarin:**Apixaban was dominant									Cost of apixaban; baseline rate of ICH; relative efficacy of ICH on apixaban compared to warfarin; long-term cost of ICH; time horizon	Apixaban was dominant 57% of the time and cost-effective 98% of the time assuming a WTP = $50,000/QALY
Lee,2012 [27]	ApixabanASA	70 year old with AF, CHADS2 score of 2 and low risk of bleeding	(2011 USD)**Compared to ASA:**ApixabanDominated in 1 year model and dominant in 10 year model									Rate of stroke on apixaban and ASA; monthly cost of major stroke; time horizon	Apixaban was cost-effective 11% of the time in the 1-year model and 96.7% of the time in the 10-year model compared to ASA assuming a WTP = $50,000/QALY
**Dabigatran vs. Rivaroxaban**													
Edwards, 2011 [28]	RivaroxabanPooled dabigatran 110/150 mgSequential dabigatranAdjusted-dose warfarinASA	Patients with AF based on the population included in the ROCKET AF trial	(2010 GBP)**Compared to sequential dabigatran:**Rivaroxaban was dominant		(2010 GBP)**Compared to dabigatran 150** **mg/110** **mg (pooled):**Rivaroxaban was dominant			(2010 GBP)**Compared to warfarin:**Rivaroxaban £18,883 ($29,576 USD)			(2010 GBP)**Compared to ASA:**Rivaroxaban £2,083 ($3,262 USD)	Time in INR range	Rivaroxaban was cost-effective 75% and 88% of the time compared to warfarin assuming WTPs = £20,000GBP/QALY and £30,000GBP/QALY, respectively
Kansal,2012 [29]	RivaroxabanSequential dabigatranAdjusted-dose warfarin	73 year old patient with AF and CHADS2 risk matched to ROCKET-AF (mean CHADS2 score of 3.5)	(2010 CAD)**Compared to rivaroxaban:**Sequential dabigatran was dominant					(2010 CAD)**Compared to warfarin:**Sequential dabigatran$6,889 ($7,071 USD)Rivaroxaban$22,475 ($23,065 USD)				None identified	Sequential dabigatran was the most cost-effective agent (98% probability) assuming a WTP = $30,000CAD/QALY
**Dabigatran vs. Rivaroxaban vs. Apixaban**													
Wells, 2012 [30]	RivaroxabanDabigatran 110 mgDabigatran 150 mgApixabanAdjusted-dose warfarin	Canadians with non-valvular atrial fibrillation with typical patient profile from the RE-LY RCT (72 years with no previous stroke or MI)	(2011 CAD)**Compared to Dabigatran 150** **mg:**Rivaroxaban was dominatedApixaban was dominatedDabigatran 110 mg was dominated		(2011 CAD)**Compared to Rivaroxaban:**Dabigatran 110 mg was dominated			(2011 CAD)**Compared to Apixaban:**Dabigatran 110 mg was dominatedRivaroxaban was dominated			(2011 CAD)**Compared to warfarin:**Dabigatran 150 mg$17,525 ($18,063 USD)Dabigatran 110 mg$96,026 ($98,975 USD)Rivaroxaban$55,757 ($57,470 USD)Apixaban$24,312 ($25,059 USD)	Baseline risk of stroke; costs of apixaban; time horizon; time in INR range; relative effects of treatments on non-vascular deaths	Dabigatran 150 mg was the optimal treatment 68.1% of the time, apixaban 29.0%, rivaroxaban 1.4%, dabigatran 110 mg 0.6% and warfarin 0.9% assuming a WTP = $50,000CAD/QALY

AF = atrial fibrillation; ASA = aspirin; CAD = Canadian dollar; CI = contraindication; ECH = extracranial hemorrhage; EUR = Euro; GI = gastrointestinal; GBP = Great Britain Pound; HR = hazard ratio; ICH = intracranial hemorrhage; INR = international normalized ratio; MCS = Monte Carlo Simulation; MI = myocardial infarction; NR = not reported; QALY = quality adjusted life year; RE-LY = Randomized Evaluation of Long-Term Anticoagulation Therapy; ROCKET-AF = Rivaroxaban Once Daily Oral Direct Factor Xa Inhibition Compared with Vitamin K Antagonism for Prevention of Stroke and Embolism Trial in Atrial Fibrillation; RR = relative risk; TIA = transient ischemic attack; TTR = time in therapeutic range; USD = United States dollar; VKA = vitamin k antagonist; WTP = willingness to pay.

a Based on a Letter to the Editor update related to overestimation of cost to dabigatran. Cost of dabigatran 150 mg twice daily reduced from $13.00/day to $8.00.

### Rivaroxaban Models

Of the four models directly comparing rivaroxaban to warfarin, three were derivatives of Sorensen et al. [16] and one of Gage et al. [34]. Similar to dabigatran, rivaroxaban models adapted from Sorensen et al. tended to be consistent in model structure and characteristics, adjusting as necessary for country specific costs, discount rates and life tables. All four models used safety and efficacy data from the ROCKET-AF trial, though base-case population characteristics varied among the four models, with three models employing hypothetical cohorts with CHADS2 risks similar to or matching patients in ROCKET-AF, and one employing a typical patient profile from RE-LY. All four models included MI and minor bleed health states, whereas the three models based on Sorensen et al. also included a systemic embolism health state. Even though all four models compared rivaroxaban to warfarin, only two of four models measured the impact of INR control on their results. In total, 3 of the 4 of rivaroxaban vs. warfarin ICERs were cost-effective at their respective WTP thresholds and ranged from $23,065–$57,470 (**Table 3** **,** **Figure 2**). Regardless, upon PSA, rivaroxaban was found to be cost-effective in at least 75% (up to 80.1%) of iterations at the lowest reported WTP thresholds. Upon one-way sensitivity analysis, results were typically sensitive to baseline rates/hazard ratios of ischemic stroke or ICH on rivaroxaban/warfarin, time horizon and the percentage of time spent in a therapeutic INR range.

### Apixaban Models

Four models included apixaban as a first line therapy for SPAF, three of which were compared to warfarin, and one compared to aspirin in a cohort of patients deemed unsuitable for warfarin. Three of the four models were adapted from Gage et al. [34], and as with the other drug models, varied in model characteristics and structure (e.g., time horizon, cycle length, health states modeled). Of note, one model based on Gage et al. modeled only patients with a prior stroke or TIA [20]. All four models included an MI health state; three modeled minor bleeding; and only one included systemic embolism as a possible health state. Of the three models comparing apixaban to warfarin, two assessed the impact of INR control on their results. In all the models comparing apixaban to warfarin, apixaban was shown to be at least a cost-effective strategy with ICERs ranging from $11,400–$25,059, if not dominant (**Table 3** **,** **Figure 2**). Upon PSA, apixaban was deemed a cost-effective strategy between 62%–98% of iterations compared to warfarin. Results of these three models were typically sensitive to changes in the cost of apixaban, baseline rates of stroke/ICH and time horizon. One model directly compared apixaban to aspirin in a hypothetical cohort of patients unsuitable for warfarin therapy. The authors chose to run two base-case analyses; one assuming a trial-length follow-up (1-year to match the mean follow-up of the AVERROES trial [35]), and one employing a longer-term (10 year) follow-up of patients. In the trial-length model, apixaban was dominated by aspirin and upon PSA was estimated to be cost-effective in only 11% of iterations. However, when a longer-time horizon was utilized, apixaban was the dominant strategy to aspirin, and was shown to be cost-effective in 96.7% of iterations at the reported WTP threshold. Results of this model were sensitive to the time horizon, rate of stroke on apixaban/aspirin and the monthly cost of major stroke upon one-way sensitivity analysis.

### Models Based Upon Indirect Treatment Comparison Meta-Analyses

Three models indirectly compared newer anticoagulants; two compared rivaroxaban to dabigatran, and one compared rivaroxaban, dabigatran and apixaban. The models derived clinical event rates using methodologies of either a mixed or indirect treatment comparison meta-analysis with warfarin as a common comparator. Data for these indirect comparisons were taken from RE-LY and PETRO, ROCKET-AF and ARISTOTLE for dabigatran, rivaroxaban and apixaban, respectively [10]–[12], [36]. Two models [28], [29] compare dabigatran and rivaroxaban outcomes based consistently on the safety-on-treatment (SOT) populations, whereas Wells et al. [30] compared dabigatran and apixaban outcomes based on the intention-to-treat (ITT) population with rivaroxaban outcomes based on both SOT and ITT populations. All three models were derivatives of Sorensen et al. [16], though two modeled a cohort of patients similar to the ROCKET-AF trial, while the third more closely matched RE-LY. Rivaroxaban was the dominant strategy compared to both sequential dabigatran and a pooled dabigatran 110 mg/150 mg strategy in one model, whereas sequential dabigatran and dabigatran 150 mg were found to be dominant strategies compared to rivaroxaban in the remaining models. Apixaban was dominated by dabigatran 150 mg, dominant compared to dabigatran 110 mg (in one model) and dominant compared to rivaroxaban (in one model), while rivaroxaban was dominant in its lone comparison versus dabigatran 110 mg. Upon PSA, one model did not report PSA results for the rivaroxaban to dabigatran comparison; while another model showed dabigatran 150 mg to be the most cost-effective agent in 68.1% of iterations, followed by apixaban (29%), rivaroxaban (1.4%), warfarin (0.9%), and dabigatran 110 mg (0.6%); and the last model showed sequential dabigatran to be the most cost-effective agent in 98% of iterations compared with rivaroxaban and warfarin (**Table 3** **,** **Figure 2**). Results of the model by Edwards et al. were sensitive to the time spent in INR range upon one-way sensitivity analysis [28]. In the comparison of dabigatran, rivaroxaban, and apixaban by Wells et al., results were also sensitive to time spent in INR range, along with the cost of apixaban, time horizon, and baseline stroke risk [30]. Interestingly, in the model by Kansal et al., dabigatran remained the preferred treatment option in all one-way sensitivity analyses performed [29].

## Discussion

There has been a rapid dissemination of newer oral anticoagulants SPAF cost-effectiveness analyses in the last few years [13]–[30]. Fourteen models evaluated dabigatran [13]–[23], [28]–[30], four evaluated rivaroxaban [24], [28]–[30] and four evaluated apixaban [25]–[27], [30]. Moreover, three models provided comparative the cost-effectiveness of two or more of the newer oral anticoagulants [28]–[30]. Six of eight models found dabigatran 150 mg to be cost effective, three of seven found dabigatran 110 mg to be cost-effective, and seven of eight found sequential dabigatran to be cost-effective versus adjusted-dose warfarin. The earlier dabigatran models generally had higher ICERs due to an over-estimation/high cost of dabigatran. Studies evaluating sequential dabigatran dosing generally showed lower ICERs than traditional dosing, although it is noteworthy that sequential dosing is not supported by the RE-LY trial and is not an approved regimen in the United States. Three apixaban models showed it to be either dominant [26] or cost-effective compared with warfarin [25], [30], whereas compared to aspirin, apixaban was dominated in a 1-year trial length model, but dominant in a longer 10-year model [27]. Commonly reported sensitive or influential variables included the cost of the newer agents, the rates of stroke/ICH versus various comparators, the time horizon, the quality of warfarin control and the costs of acute events and long term disability care.

One of the challenges in attempting to evaluate the comparative cost-effectiveness of newer oral anticoagulants is the difficulty in making cross-model comparisons. This is likely true in the case of these newer SPAF models, even though a majority of them used the basic and common structures of Gage [34] or Sorensen [16]. This is because the models had some differences in health states included, made different assumptions and used varying inputs. In some instances, similar models were performed from the perspective of varying countries, this was necessary in order to not only address differences in costs, discount rates and average life spans (life tables), but also to address the varying approved dosing schemes from country-to-country (i.e., sequentially-dosed dabigatran is not an FDA approved regimen). Three models used data from either adjusted indirect comparison meta-analyses or network meta-analyses [28]–[30]; however, even the results of these models must be interpreted with caution due to important differences in the studies that underlie the comparisons and the conduction of the indirect comparisons themselves. Of importance, the 3 major clinical trials evaluating the newer oral anticoagulant agents vs. warfarin differ in notable ways [10]–[12].The ROCKET-AF trial enrolled patients at higher baseline ischemic stroke risk than the RE-LY or ARISTOTLE trials, with mean CHADS_2_ scores of 3.5, 2.1, and 2.1, respectively. In addition, the quality of warfarin dosing was not consistent across studies with patients spending less time within the therapeutic INR range in ROCKET-AF (55%) versus either RE-LY (64%) or ARISTOTLE (62%). In fact, methodological guidance documents would suggest this may be an inappropriate situation for indirect comparison due to the lack of comparability/heterogeneity of the trials to be pooled [37]–[39]. Also, as alluded to previously, endpoint data used both within and across the indirect comparisons were not always based on the same trial populations/analysis methods, some using ITT populations and others using SOT populations. Thus, it is not surprising that these indirect comparison meta-analyses had disparate effect size estimates for many of the key model inputs [29], [30], [40]–[42]. In 5 identified meta-analyses making indirect comparison of at least 2 of the newer agents, marked variation in relative effect size estimates can be observed. For example, odds ratios of dabigatran versus rivaroxaban ranged from: 0.74–0.85 for stroke/systemic embolism, 0.95–1.06 for all-cause mortality, and 1.59–1.76 for acute MI. Similarly hazard ratios ranged from 0.96–1.04 for all-cause mortality, 1.40–1.57 for acute MI and 0.48–0.63 for ICH.

Importantly, all of the identified models in this review utilized a lone RCT (or an indirect comparison in which only a lone study existed for a given direct comparison) to characterize the main efficacy and safety comparisons between treatments. Data from these short-term clinical trials had to be extrapolated to longer time horizons in order to estimate the cost-effectiveness of agents. While in theory, conducting a piggy-backed economic analysis alongside a substantially longer RCT would yield more rigorous results, this would be both time and cost prohibitive. Thus, this limitation of the underlying trials leads to the greatest asset of models; that is, they systematically allow for extrapolation of data to provide decision-makers with some, albeit not perfect, data to make necessary coverage decisions. In addition, while these extrapolations involve generalizations and assumptions, modeling provides a way of systematically managing uncertainty and assessing the impact of these assumptions on the results through sensitivity analyses [43], [44].

The lack of standardized guidelines for conducting economic analyses poses problems in the accurate validity assessment, and therefore interpretation of the results and conclusions of these analyses. The use of outdated non-drug specific may reduce the validity of some of these models. Variations in the inclusion of health states, even across models assessing similar drugs, also presents difficulties in translating results, especially in cases of disagreement in the conclusions of those models. Decision makers must be aware of these caveats when clinical and coverage decisions are formed on the basis of these economic analyses.

### Conclusions

Many researchers have published cost-effectiveness models of the novel anticoagulants for SPAF. These models suggest that the novel anticoagulants are cost-effective, but do not provide adequate data for direct comparison of the individual agents. For now, it seems prudent to choose anticoagulation therapy on a patient-specific basis. Standardization of the structure and inputs to assure that important health states are not being ignored and the best and most recent inputs are utilized would improve future comparisons between SPAF models. In addition, head-to-head trials of the newer oral anticoagulants would aid health economists to assess their comparative cost-effectiveness.

## Supporting Information

Figure S1 **Pictorial Comparison of Sorensen (A) and Gage (B) Models.**
(TIF)

Figure S2 **Proportion of Models Utilizing Specified Treatment Arm.** *Any warfarin treatment arm: standard care warfarin; genotype-guided warfarin; “perfect” warfarin; “trial-like” warfarin; or “real-world prescribing” warfarin †Any dabigatran treatment arm: 110 mg; 150 mg; or sequential.
(TIF)

Figure S3 **Primary Comparison.**
(TIF)

Figure S4 **Quality of Health Economic Studies.** Refer to Appendix Text 2 for interpretation of QHES scoring criteria.
(TIF)

Text S1 **MEDLINE Search Strategy.**
(DOCX)

Text S2 **Explanation of Quality of Health Economic Studies (QHES) Scoring of Included Models.**
(DOCX)

Checklist S1 **PRSIMA 2009 Statement Checklist.**
(DOC)
