# Supplementary figures and images for: Novel Anticoagulants for Stroke Prevention in Atrial Fibrillation: A Systematic Review of Cost-Effectiveness Models
Source: PLoS One. 2013 Apr 23;8(4):e62183. doi: 10.1371/journal.pone.0062183 (PMC3633898; doi:10.1371/journal.pone.0062183)

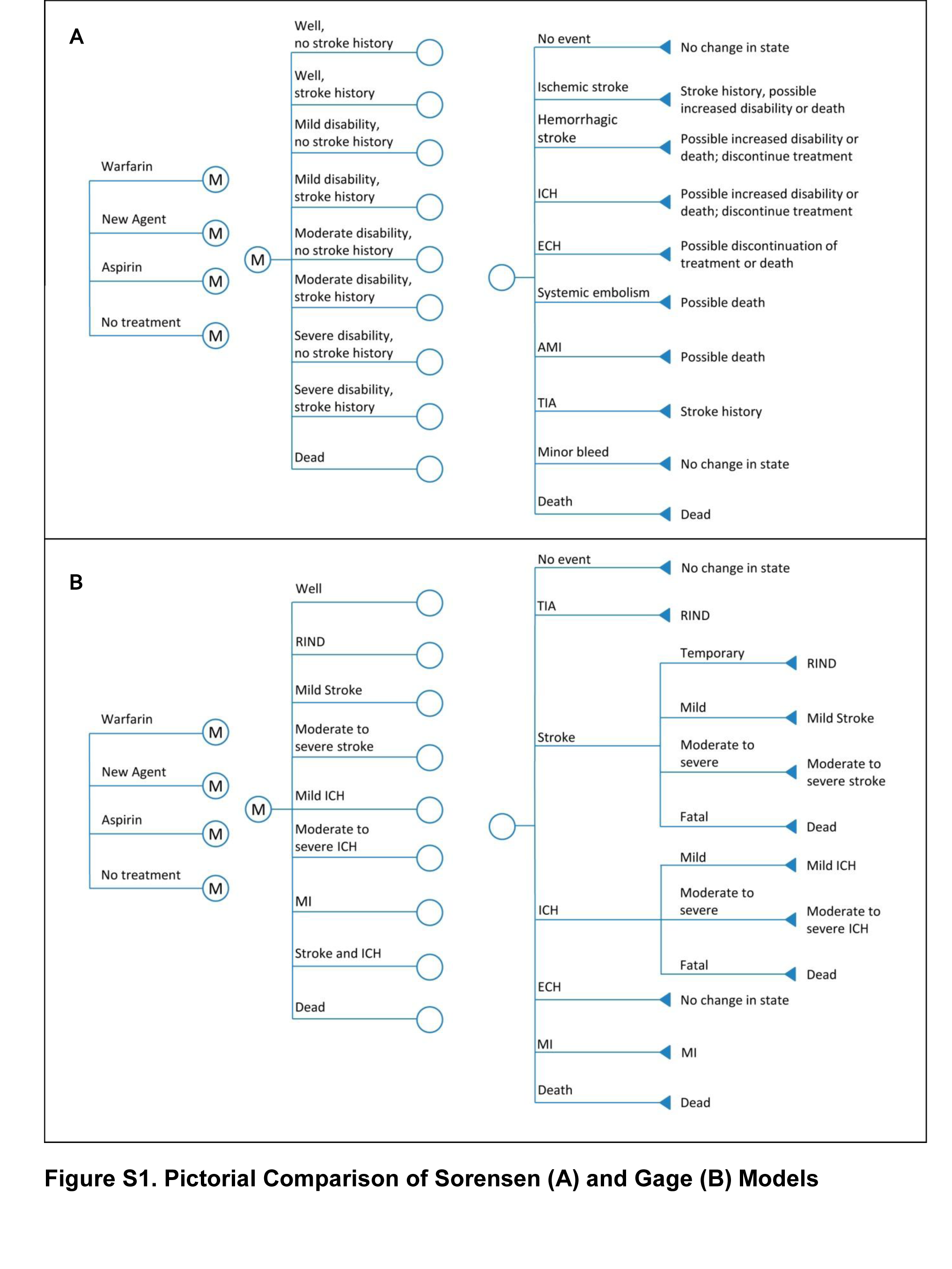

Supplement: Figure S1 — Pictorial Comparison of Sorensen (A) and Gage (B) Models. (TIF) [file pone.0062183.s001.tif]

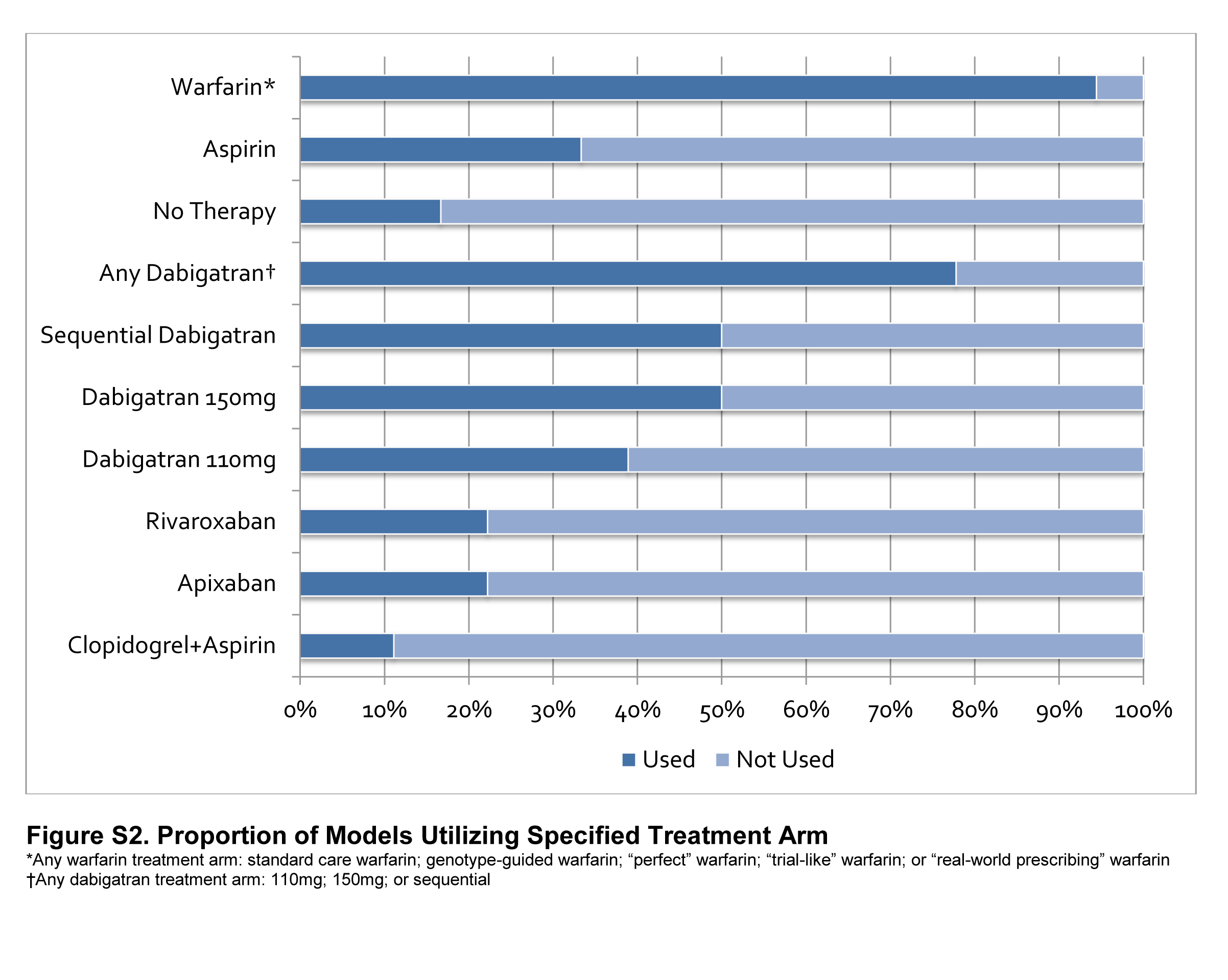

Supplement: Figure S2 — Proportion of Models Utilizing Specified Treatment Arm. *Any warfarin treatment arm: standard care warfarin; genotype-guided warfarin; “perfect” warfarin; “trial-like” warfarin; or “real-world prescribing” warfarin †Any dabigatran treatment arm: 110 mg; 150 mg; or sequential. (TIF) [file pone.0062183.s002.tif]

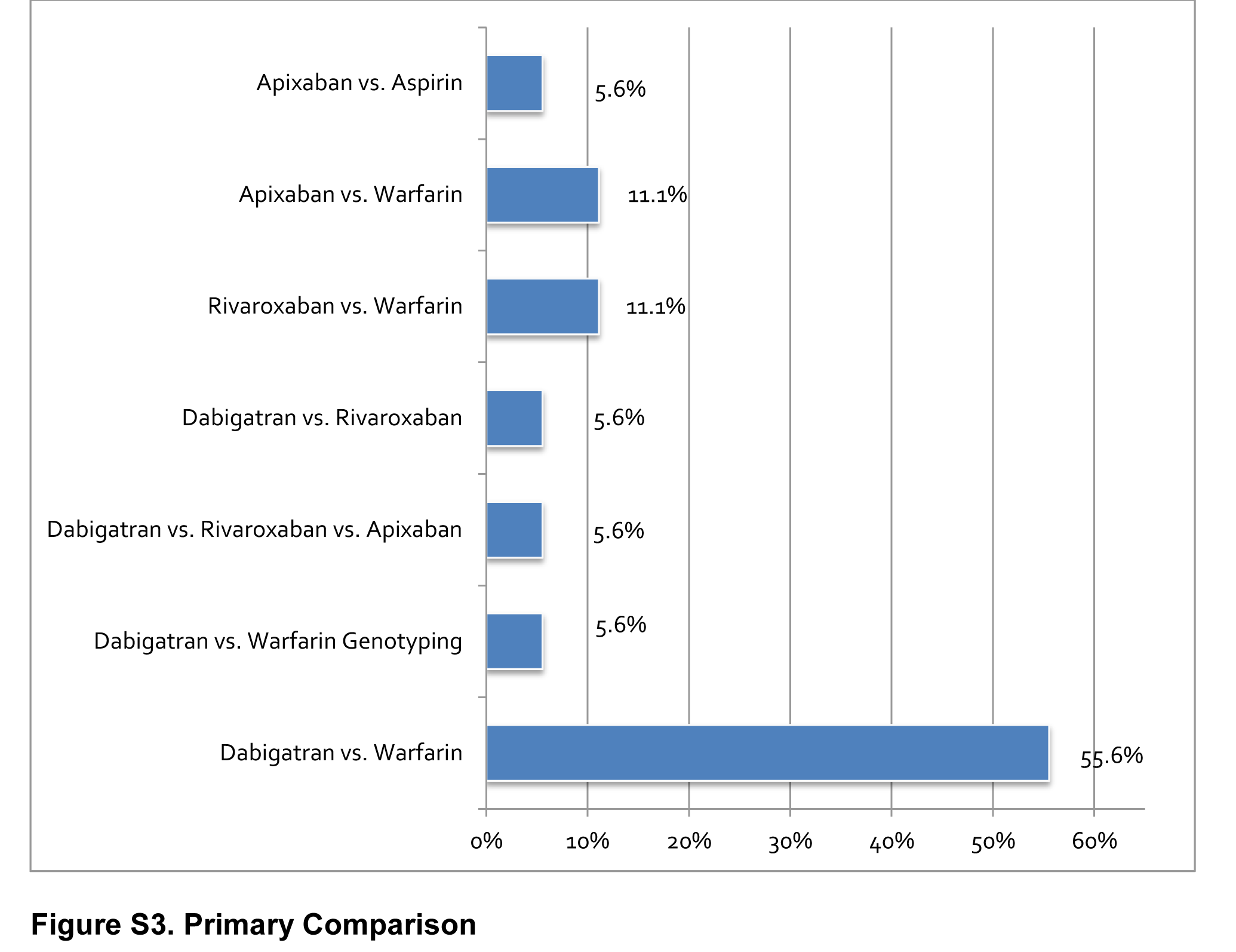

Supplement: Figure S3 — Primary Comparison. (TIF) [file pone.0062183.s003.tif]

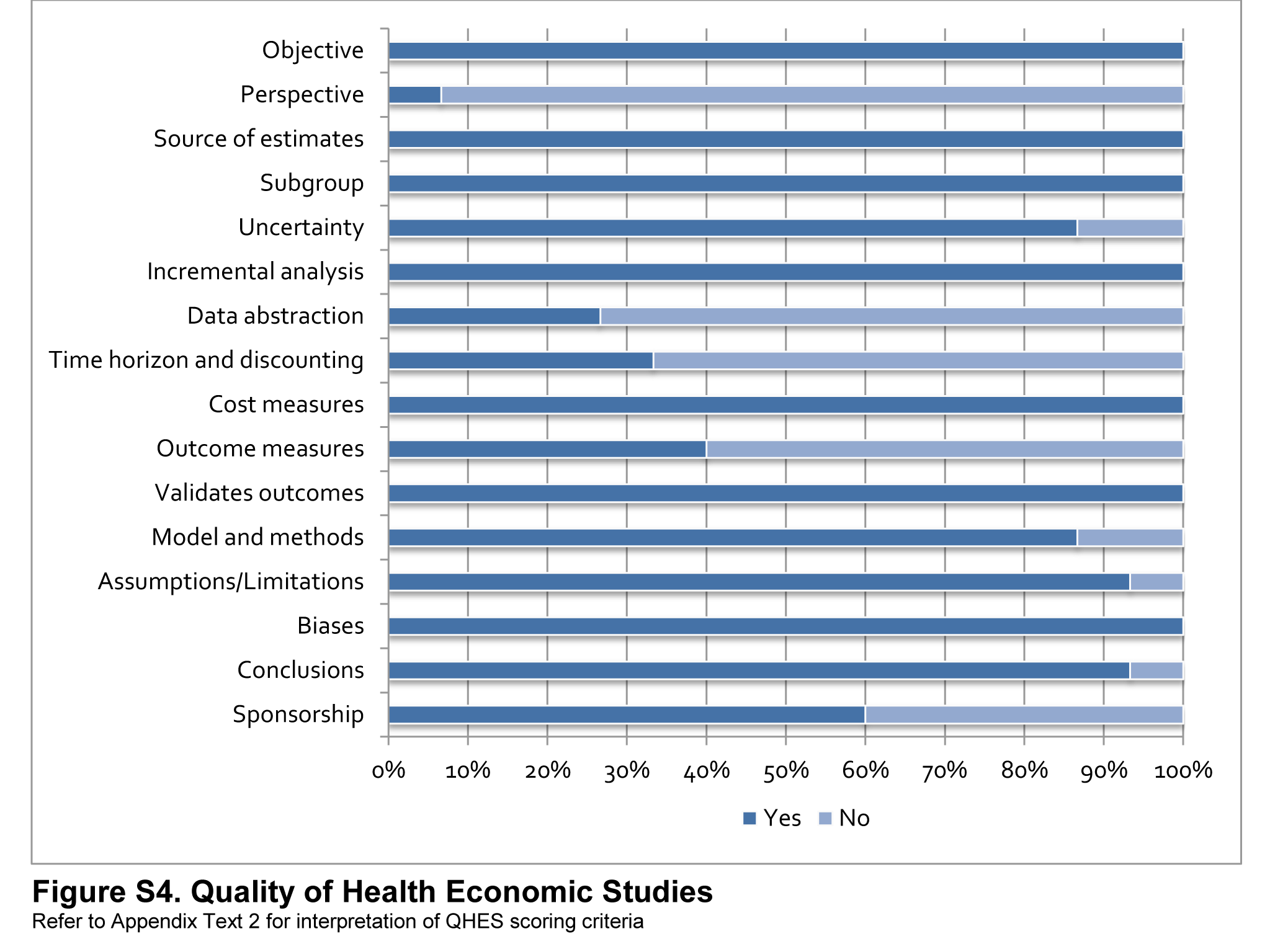

Supplement: Figure S4 — Quality of Health Economic Studies. Refer to Appendix Text 2 for interpretation of QHES scoring criteria. (TIF) [file pone.0062183.s004.tif]
