# Supplementary material for: Novel Anticoagulants for Stroke Prevention in Atrial Fibrillation: A Systematic Review of Cost-Effectiveness Models
Source: PLoS One. 2013 Apr 23;8(4):e62183. doi: 10.1371/journal.pone.0062183 (PMC3633898; doi:10.1371/journal.pone.0062183)
Supplement: Text S1 — MEDLINE Search Strategy. (DOCX) [file pone.0062183.s005.docx]

**Text S1: MEDLINE Search Strategy**

Atrial fibrillation AND (markov OR semi-markov OR markov state transition model OR markov simulation OR markov chain OR markov processes OR decision analysis OR decision analyses OR decision analytic OR decision tree OR decision model) AND (warfarin OR coumarins OR vitamin K antagonists OR dabigatran OR apixaban OR rivaroxaban OR ximelagatran OR anticoagulants OR aspirin OR clopidogrel OR antiplatelet)
