# Supplementary material for: Novel Anticoagulants for Stroke Prevention in Atrial Fibrillation: A Systematic Review of Cost-Effectiveness Models
Source: PLoS One. 2013 Apr 23;8(4):e62183. doi: 10.1371/journal.pone.0062183 (PMC3633898; doi:10.1371/journal.pone.0062183)
Supplement: Text S2 — Explanation of Quality of Health Economic Studies (QHES) Scoring of Included Models. (DOCX) [file pone.0062183.s006.docx]

**Text S2: Explanation of Quality of Health Economic Studies (QHES) Scoring of Included Models**

1. **Was the study objective presented in a clear, specific, and measurable manner? (7 points)**

Self-explanatory. The study objective did not have to be stated/described in a single statement or be located in a specific section of the paper.

1. **Were the perspective of the analysis (societal, third-party payer, etc.) and reasons for its selection stated? (4 points)**

Scored “yes” if the perspective was stated and the perspective stated matched the cost and consequence inputs used in the analysis. Models using the societal perspective had to include direct and indirect costs and measure QALYs. An explanation for using the societal perspective was not required (since it is the broadest/most comprehensive perspective). Justification for using a perspective other than societal had to be given to be scored a “yes”. The perspective justification study objective did not have to be stated/described in a single statement or be located in a specific section of the paper.

1. **Were variable estimates used in the analysis from the best available (i.e. randomized control trial - best, expert opinion - worst)? (8 points)**

Scored “yes” if the best available (most internally valid) source of data for underlying model assumptions of efficacy and safety were used. When available, well done meta-analyses/pooled analyses should have been used for sources in place of single randomized trials.

1. **If estimates came from a subgroup analysis, were the groups pre-specified at the beginning of the study? (1 point)**

Scored “yes” if the estimate came from a source pre-specified at the beginning of the study or if no subgroup analysis was performed; scored “no” if there was no indication that the subgroup analysis was planned prior to the start of the economic evaluation.

1. **Was uncertainty handled by (1) statistical analysis to address random events, (2) sensitivity analysis to cover a range of assumptions? (9 points)**

Scored “yes” if deterministic sensitivity analysis (i.e., one- or multi-way sensitivity analysis) AND probabilistic sensitivity analysis (i.e., Monte Carlo simulation) were conducted and results of both were reported.

1. **Was incremental analysis performed between alternatives for resources and costs? (6 points)**

Scored “yes” if an incremental cost-effectiveness ratio (ICER) was reported, or if a therapy was found to be dominant, the costs and benefits of each intervention were presented and dominance was clearly stated.

1. **Was the methodology for data abstraction (including the value of health states and other benefits) stated? (5 points)**

Scored “yes” if values for all health states and/or other benefits were stated AND their means of identification were described (i.e., a systematic search of the literature)

1. **Did the analytic horizon allow time for all relevant and important outcomes? Were benefits and costs that went beyond 1 year discounted and justification given for the discount rate? (7 points)**

Scored “yes” if 1) the time horizon used in the model was at least 10 years in duration, 2) the cohort was followed until at least 75 years of age (stating the cohort was followed for a lifetime was considered sufficient to meet the second requirement), 3) the discount rate applied was clearly stated, and (4) the specific discount rate was justified (simply providing a reference for the discount rate was not considered sufficient to fulfill the last requirement).

1. **Was the measurement of costs appropriate and the methodology for the estimation of quantities and unit costs clearly described? (8 points)**

Scored “yes” if all relevant costs and consequences for each alternative were identified, valued appropriately, and sources of values described.

1. **Were the primary outcome measure(s) for the economic evaluation clearly stated and were the major short-term, long-term, and negative outcomes included? (6 points)**

Scored “yes” if 1) a clear statement of primary outcome measure was made (i.e., cost per quality-adjusted life-year (QALY) or life-year (LY)), 2) all important negative outcomes (adverse events) were included the analysis. To meet this latter requirement, all models had to include ischemic stroke, intracranial bleeding, major extracranial bleeding and minor bleeding as health states. When underlying clinical trial data suggested a statistically significant difference in other negative outcomes (i.e., myocardial infarction, dyspepsia), these had to be included as health states in the model as well. Systemic embolism was not required to be a health state in any model.

1. **Were the health outcomes measures/scales valid and reliable? If previously tested valid and reliable measures were not available, was justification given for the measures/scales used? (7 points)**

Scored “yes” if a valid and reliable sources of health utility values were used OR if such sources did not exist, justification for an alternative source or not utilizing the utility value was given.

1. **Were the economic model (including structure), study methods and analysis, and the components of the numerator and denominator (cost/QALY or LY) displayed in a clear, transparent manner? (8 points)**

Scored “yes” if 1) a clear and transparent display of the economic model (including structure) was provided, OR if using the same structure as a previously published model, referring readers to the model in a previous paper was considered sufficient as long as no change to the model structure was reported, 2) the model’s methods for estimating both the numerator (costs) and denominator (QALYs/LYs) were displayed in a clear, transparent manner, and 3) numerator (costs) and denominator (QALYs/LYs) results were presented in a clear and transparent manner.

1. **Were the choice of economic model, main assumptions, and limitations of the study stated and justified? (7 points)**

Scored “yes” if the main assumptions and limitations of the model were stated and justified.

1. **Did the author(s) explicitly discuss direction and magnitude of potential biases? (6 points)**

Scored “yes” if potential biases were mentioned (including the direction of the bias) AND the authors attempted to explain the biases.

1. **Were the conclusions/recommendations of the study justified and based on the study results? (8 points)**

Scored “yes” if 1) the conclusions/recommendations of the study were in agreement with the results reported in the paper (*note: conclusions about cost-effectiveness cannot be made unless an incremental cost-effectiveness ratio (ICER) was calculated (except in the case of dominance) AND a reasonable willingness-to-pay threshold was stated, and 2) when possible, the results of the model were compared with others addressing the same economic question.

1. **Was there a statement disclosing the source of funding for the study? (3 points)**

Score “yes” if the funding source was provided OR the absence of funding was noted. Scored “no” if no statement of funding (or lack thereof) was provided.
